# Direct Extraction of Uranium Oxides with *N,N*-Di(2-ethylhexyl)isobutyramide

**DOI:** 10.1021/acsomega.5c01103

**Published:** 2025-03-28

**Authors:** Amy L. Speelman, Daria Boglaienko, Avalon B. Tarbet-Mendoza, Nathan P. Bessen, Ashley N. Williams, Sergey I. Sinkov, Bruce K. McNamara, Gregg J. Lumetta, Gabriel B. Hall

**Affiliations:** Pacific Northwest National Laboratory, Richland, Washington 99352, United States

## Abstract

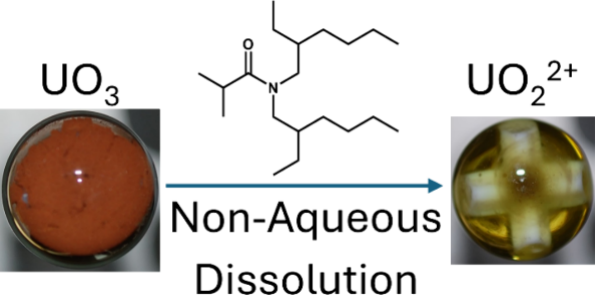

The direct extraction of uranium from voloxidized nuclear fuel into an organic solvent offers several potential advantages over conventional hydrometallurgical reprocessing, including reducing the reprocessing plant footprint, providing an initial degree of decontamination from fission products, and minimizing the amount of secondary waste from nitric acid. In this work, the direct extraction of uranium oxides into 1.5 M *N,N*-di(2-ethylhexyl)isobutyramide (DEHiBA) in *n-*dodecane is examined. UV–vis spectra and distribution ratios of HNO_2_ in 1.5 M DEHiBA as well as the equilibrium organic phase H_2_O concentrations in HNO_3_-loaded 1.5 M DEHiBA are also reported. Hypothesized reaction stoichiometries for the direct extraction of uranium from UO_2_, α-U_3_O_8_, and ε-UO_3_ are verified through analysis of organic-phase U, HNO_2_, and HNO_3_ concentrations after dissolution. Water generated by the dissolution results in the formation of a separate aqueous phase, which will need to be accounted for in future flowsheet design.

## 1 Introduction

Used nuclear fuel (UNF) is traditionally reprocessed by first dissolving the fuel matrix in hot nitric acid (HNO_3_) and then using solvent extraction to recover valuable components. Since nearly all the elements in UNF dissolve in hot HNO_3_, the burden of separating valuable components from undesired components is placed entirely on solvent extraction. Additionally, the use of hot concentrated HNO_3_ necessitates a complicated off-gas abatement system. Significant process intensification could be achieved by instead directly dissolving the UNF into the process solvent, eliminating the equipment and infrastructure associated with hot aqueous HNO_3_ dissolution. The generation of secondary nuclear waste will also be minimized due to reduced HNO_3_ requirements. Furthermore, many of the fission products for which the process solvent has low affinity could potentially remain as undissolved solids, which would provide an initial degree of decontamination of the desired products from the fission products.

The concept of direct extraction of UNF was first introduced in 1966 in a patent by Tomijima et al.^1^ but has received relatively little attention since. The initial work by Tomijima focused on tributyl phosphate (TBP) as the extracting agent. More recent studies also use TBP in an organic diluent^2−9^ or in liquid or supercritical CO_2_ (RELICT^10,11^ and Super-DIREX processes^12−16^). However, an important tenet that has underscored nuclear fuel processing research and development over the last several decades is the CHON principle,^17^ which requires extractant molecules to consist of only the elements carbon (C), hydrogen (H), oxygen (O), and nitrogen (N). Such molecules can be disposed of at the end of their useful life by thermal decomposition yielding only gaseous byproducts, thus avoiding the creation of a solid secondary waste stream. Since TBP does not conform to the CHON principle, alternative extractants are sought for direct extraction of UNF.

**Figure 1:**
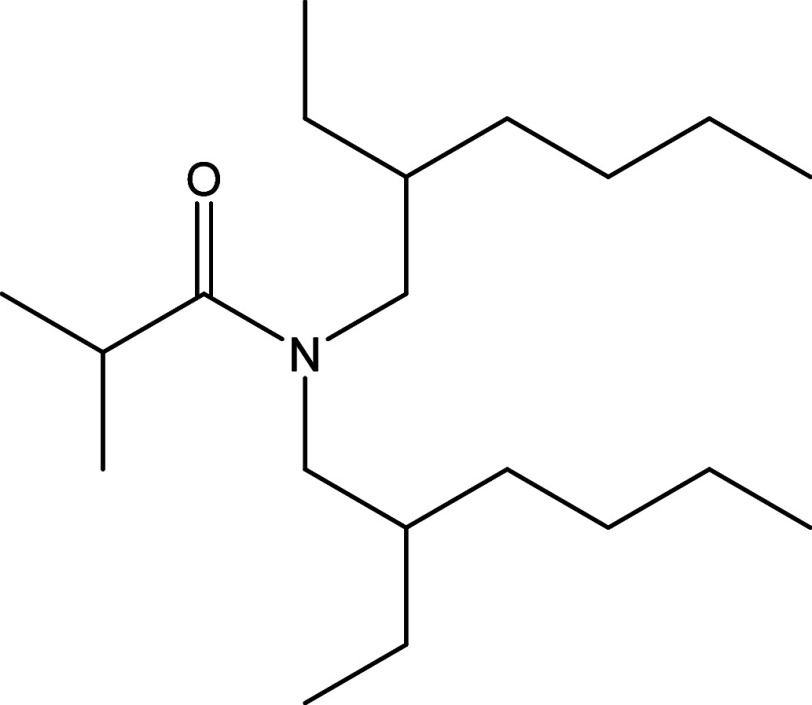
Chemical structure of DEHiBA.

Dialkylamides are CHON-compliant extractants that have relatively benign hydrolysis and radiolysis products.^18,19^ Among the dialkylamides, *N,N*-di(2-ethylhexyl)isobutyramide (DEHiBA, Figure 1) has received considerable attention because of its high selectivity for hexavalent actinides, particularly uranium(VI).^20^ This property is important when isolation of a pure uranium (U) stream is desirable, for example during recovery of U with residual enrichment from high assay low enriched uranium (HALEU) fuel. The use of DEHiBA for extraction of U from dissolved irradiated fuel has been demonstrated in counter-current solvent extraction tests.^21,22^ Prior work in our laboratory has focused on intensifying the process by increasing the solvent loading for U through increasing the DEHiBA concentration from 1.0 to 1.5 M.^23,24^ In the current work, the direct extraction of U into 1.5 M DEHiBA is examined as a means to further intensify the process of U extraction from UNF.

An additional consideration in this work is that it is assumed a volumetric oxidation (voloxidation) step will be conducted upstream from the direct extraction. Voloxidation offers several advantages over conventional front-end treatment of UNF and is viewed as a likely operation in future UNF processing plants in the United States (US). The primary motivation to implement voloxidation is to release volatile fission products prior to fuel dissolution so that they can be conveniently captured in a compact off-gas treatment system, thereby reducing the plant capital and operational costs. When air or oxygen is used as oxidant, tritium is released from the fuel matrix during voloxidation.^25^ However, iodine is also reported to be removed if NO_2_ is used as the oxidant, which would offer a significant operational advantage in managing this problematic fission product.^26^

The primary U-containing product from NO_2_ voloxidation is ε-UO_3_,^27^ whereas α-U_3_O_8_ is formed when oxygen is used as oxidant.^28^ Conversion to these higher U oxides results in lattice cell expansion, which causes the fuel to fragment into a powder. This should result in faster dissolution due to the higher surface area of the powder. The kinetic component of dissolution will be particularly important for direct extraction due to the increased time the solvent may need to spend in a high-dose field. While some radioprotective effect has been observed for DEHiBA loaded with U,^29^ it is desirable to achieve the lowest possible residence time of the solvent with high dose contributors which are expected to remain as undissolved solids.

Because of the advantages discussed above, research and development efforts in the US currently focus on advanced voloxidation with NO_2_, which progresses according to the following reactions:^27^
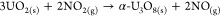
1
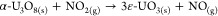
2

Further conversion of ε-UO_3_ to UO_2_(NO)(NO_3_)_3_ or UO_2_(NO_3_)_2_ has also been reported, but the conditions leading to these products are less well-understood.^30^ The present effort therefore focuses on investigating direct extraction of U from α-U_3_O_8_ and ε-UO_3_, along with UO_2_ which may be present due to incomplete oxidation.

In a direct extraction process, the solvent is first loaded with HNO_3_ which is required for conversion of the U oxides to soluble uranyl nitrate. Hypothetical reaction stoichiometries can be proposed based on balancing equations involved in dissolution:
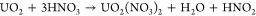
3
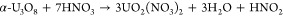
4
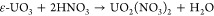
5

In reactions 3 and 4, the HNO_3_ acts as an oxidant for the low-valent U present in UO_2_ and U_3_O_8_ leading to the production of nitrogen oxide (NO_*x*_) byproducts which are written as HNO_2_, although NO and/or NO_2_ may also be produced. In addition to NO_*x*_, the reactions are expected to produce H_2_O which could either remain in the organic phase or form a separate aqueous phase. Since no oxidation of U occurs in reaction 5, no NO_*x*_ generation is anticipated, but again the water that is formed may produce a separated aqueous phase. The work reported here is directed at demonstrating direct extraction of U oxides with DEHiBA and validating the hypothetical reaction stoichiometries in eqs 3-5. The resulting information about the consumption of HNO_3_ as well as the production of H_2_O and HNO_2_ will support further maturation of direct extraction technology.

## 2 Experimental Methods

### 2.1 Materials

Aqueous HNO_3_ solutions were prepared by diluting concentrated (∼15 M) HNO_3_ with deionized water. The final HNO_3_ concentrations were determined by potentiometric titration with standardized NaOH solution as described below. The 1.5 M DEHiBA in *n*-dodecane stock solution used in these experiments was prepared by diluting the desired mass of DEHiBA (Marshallton, 99%) with *n*-dodecane (Sigma-Aldrich, ≥ 99%) in a volumetric flask. Except where noted otherwise, 1.5 M DEHiBA solutions were pre-equilibrated with HNO_3_ by contacting twice with an equal volume of aqueous HNO_3_ for 20 min at room temperature on a vortex mixer. All other chemicals were used as received from commercial suppliers.

### 2.2 Preparation of U Oxides

The U oxide starting material was procured as U_3_O_8_ from International Bio-Analytical Industries, Inc. (FL USA); however, powder X-ray diffraction analysis of this material showed it to be primarily a uranyl hydroxide phase (Figure S1). This material was first converted to UO_2_, then to α-U_3_O_8_ and ε-UO_3_. To produce UO_2_, approximately 9 g of the as-received powder was placed in an alumina boat inside of a steel tube of a split tube furnace (OTF-1200X, MTI Corporation, CA USA) and heated to 650 °C under a flow of 4% H_2_ in N_2_ for 9 h. Part of the obtained UO_2_ powder was used to synthesize α-U_3_O_8_ under O_2_ flow at 440 °C for around 67 h. Half of the resulting α-U_3_O_8_ powder was treated with an O_3_ in O_2_ flow at 230 °C for 20 h to synthesize ε-UO_3_.^31^ The O_3_ in O_2_ flow was generated by passing O_2_ through an ozone generator from Ozone solutions (AZCO Industries LTD, Canada) at a flow rate of approximately 1.5 L/min.

### 2.3 Powder X-ray Diffraction (PXRD)

Samples of each U oxide were loaded into Bruker manufactured sample holders. Each sample was covered with a Kapton film for radiological containment. The diffraction data was collected using a Rigaku Ultima IV Diffractometer (Cu–Kα radiation). The scans were collected in 0.02° count binning steps at a scan rate of 3° min^–1^. Data was collected from 5 to 60° 2θ using a 5 mm divergence slit, 0.5 mm incident slit, and 5° receiving side solar slits, and a Ni foil filter was used to reduce contributions from Kβ radiation. The data were analyzed and compared against reference PXRD patterns using the DIFFRAC-EVA 6.0 software package (Bruker Analytical X-ray Systems, 2021).

### 2.4 Analysis of U Oxidation States by H_3_PO_4_ Digestion

A small amount of U oxide material (typically 20 to 40 mg) was treated with 1 mL of 85% H_3_PO_4_ containing 0.141 M Na_2_SO_4_ under heating at near boiling temperature of the solvent (∼150 °C) until complete dissolution was achieved. The fully digested sample was filtered (0.45 μm pore size syringe filter) and its optical absorbance spectrum was measured against an H_3_PO_4_–Na_2_SO_4_ blank. The optical absorbances at 420 nm (U(VI) peak maximum) and 667 nm (U(IV) peak maximum) were quickly evaluated and the sample was diluted if the optical absorbance at one or both wavelengths exceeded 1.5 units. The molar concentrations of U(VI) and U(IV) were calculated from the peak maxima of the spectrum using the following molar absorptivities: ε_420 nm_ = 11.96 M^–1^cm^–1^ for U(VI), ε_420 nm_ = 6.65 M^–1^cm^–1^ for U(IV), and ε_667 nm_ = 51.29 M^–1^cm^–1^ for U(IV).

### 2.5 Raman Spectroscopy

Raman spectra of the ε-UO_3_ powder were collected through the bottom of a glass vial in three different areas of the vial using an InPhotonic spectrometer with a 670.39 nm excitation wavelength. The spectra were processed using Thermo Fisher OMNIC software and then averaged.

### 2.6 X-ray Absorption Spectroscopy

X-ray absorption spectra (XAS), i.e. U L_3_-edge X-ray absorption near edge spectroscopy (XANES), data were collected using a laboratory-based easyXAFS300 spectrometer in transmission mode.^32^ Uranium L_3_-edge spectra were collected with a Si(1266) spherically bent crystal analyzer and an Ag anode X-ray tube at 35 kV voltage and 28 mA current.

Samples were prepared by mixing 18–20 mg of the UO_2_, α-U_3_O_8_, or ε-UO_3_ powders with boron nitride filler and cellulose binder and pressing into 13 mm pellets using a 2-ton manual press within a radiological fume hood. Each pellet was wrapped in a polyamide tape to maintain its integrity. The energy calibration of the spectrometer was performed using a zirconium foil standard (procured from Exafs Materials, CA) and compared to a zirconium foil spectrum collected at NSLS X11B retrieved from the Hephaestus standards database (Demeter system, version 0.9.26).^33^ The number of scans per sample and per background spectrum were the same (70 scans, 40 min each). The silicon drift detector deadtime was kept below 30%. All the spectra were deadtime corrected, and theta-to-energy correction was applied to all the data sets, using theta-to-energy nonlinear shift of the zirconium standard foil. XAS data processing and data normalization was performed using the Athena program (Demeter system, version 0.9.26).^33^

### 2.7 Potentiometric Titration

Potentiometric titrations were performed using a Metrohm autotitrator system with a 905 Titrando controller and an 805 Dosino dosing device. Titrations were performed with 0.1 M NaOH that had been standardized with potassium hydrogen phthalate. Potentiometric titrations of the organic phase before and after U loading were performed by adding 0.2–0.4 mL aliquots of the organic phase to 25 mL of 0.2 M ammonium oxalate in deionized water. This matrix was selected in order to prevent uranium hydrolysis.^34^ The samples were mixed with a magnetic stir bar throughout the titration. All other titrations were performed in deionized water. The uncertainty in HNO_3_ concentration was either calculated by propagation of error from the uncertainties in the sample volume, NaOH concentration, and NaOH volume or taken as the standard deviation of replicate measurements, whichever value was larger.

### 2.8 UV–vis Spectroscopy

Ultraviolet–visible (UV–vis) absorption spectra of HNO_2_ were recorded using a Cary 6000i UV–vis-NIR instrument. Spectra of U-containing solutions were recorded on an SI Photonics Model 440 CCD Array UV–vis spectrophotometer equipped with fiber optic cables (Ocean Insight, P600–2-VIS-NIR) fed into a radiological fume hood. All spectral data were corrected for baseline offsets based on a linear fit of the absorbance as a function of wavelength in a featureless region of the spectrum.

To allow deconvolution of U and HNO_2_ signals in the direct extraction samples, solutions of each species in 1.5 M DEHiBA were generated and spectra were recorded on the SI Photonics UV–vis spectrophotometer. The 1.5 M DEHiBA used for these experiments was precontacted twice with 5 M HNO_3_ which results in an organic-phase HNO_3_ concentration that is approximately the same as the HNO_3_ concentrations in the UV–vis samples of U-loaded solutions from the direct extraction experiments.

To generate a U in DEHiBA solution, an aqueous solution containing 0.11 M UO_2_(NO_3_)_2_ in 5 M HNO_3_ was prepared from a legacy UO_2_(NO_3_)_2_ stock solution. A UV–vis spectrum was measured to confirm the starting U concentration using U(VI) molar absorptivities reported in literature.^35^ An aliquot of this solution was then combined with an equal volume of 1.5 M DEHiBA and contacted for 20 min on a shaker plate. The sample was centrifuged and the aqueous and organic phases were collected and analyzed by UV–vis spectroscopy. The decrease in the maximum U absorbance in the aqueous phase after the contact was used to determine the concentration of U in the organic phase. The U concentrations determined using this approach were further confirmed by ICP-OES analysis. To account for differences in photometric accuracy and wavelength calibration between the Cary and SI Photonics instruments, a solution of HNO_2_ in 1.5 M DEHiBA was prepared following the procedure in Section 2.12 and spectra were recorded on both instruments.

The U and HNO_2_ concentrations in the direct extraction samples were determined by solving a system of linear equations based on the absorbances of the sample and the reference spectra at 389 nm (HNO_2_ peak) and 423 nm (U peak). The uncertainty in the concentrations determined from this approach is assumed to be 10%.

### 2.9 Karl Fischer Titration

Portions of a 1.5 M DEHiBA in *n*-dodecane stock solution were contacted once with an equal volume of aqueous HNO_3_ for 20 min at 25 °C on a Benchmark MultiTherm shaker at 1500 rpm. The water content of the resulting organic phases was determined using a Metrohm Eco coulometer equipped with a generator electrode with a diaphragm. The anolyte was 100 mL of Hydranal Coulomat AG-H containing 20 g of imidazole and the catholyte was Hydranal Coulomat CG. The water content of the samples was determined by injecting samples into the titrator using a syringe. The mass of added sample was converted to a volume based on densities determined by weighing 200 μL aliquots of the same solutions dispensed by a calibrated micropipette. Contacts were performed in duplicate. The reported error is the difference between the two replicates.

Karl Fischer titration of U-containing samples was performed using a Photovolt Aquatest 2010 moisture analyzer equipped with a generator electrode with a diaphragm. The anolyte and catholyte are the same as those used in nonradiological titrations. The titrator vessel was opened, 200 μL samples were quickly added using a micropipette, and the titrator vessel was immediately closed. While this procedure can result in absolute H_2_O concentrations that are biased high due to the introduction of atmospheric moisture, the error is systematic and the difference in water content between two samples (e.g., before and after U dissolution) can still be determined accurately if both samples are analyzed on the same day using the same procedure. For this reason, a sample of the batch of HNO_3_-loaded 1.5 M DEHiBA used for the direct extraction was analyzed at the same time as the U-loaded solvent from that extraction. The samples were analyzed in triplicate, and the reported error is the standard deviation of the 3 measurements.

### 2.10 ICP-OES Measurements

Inductively coupled plasma optical emission spectroscopy (ICP-OES) data were obtained on an Avio 220 Max ICP-OES instrument. All samples were prepared in stocks consisting of 5% v/v of 65–70% HNO_3_ (J.T. Baker Instra-Analyzed for trace metal analysis) in deionized water. Calibration solutions were prepared from a 10,000 ppm U standard solution (Inorganic Ventures). Uranium was analyzed in axial view at 385.958 nm. The reported value is the average of 4 total measurements (2 measurements performed on 2 independently prepared samples). The uncertainty in U concentration is assumed to be 10%.

### 2.11 Procedure for Griess Assays^36^

Samples were diluted with deionized water to give final nitrite concentrations in the 1–10 μM range. A set of calibration samples were prepared by diluting a stock solution of sodium nitrite to the same concentration range. Reagent solutions containing 10 mg/mL sulfanilic acid in 5% v/v 85% H_3_PO_4_ and 1 mg/mL *N*-(1-naphthyl)ethylenediamine dihydrochloride (NEDD) in deionized water were prepared fresh each day. The assay was performed by adding 50 μL of sulfanilic acid solution followed by 50 μL of NEDD solution to 2.9 mL of sample. After allowing color to develop for 30 min, UV–vis spectra were recorded and nitrite concentrations were determined based on comparison of the absorbance at 545 nm to the calibration curve. The uncertainty in HNO_2_ concentration is assumed to be 10%.

### 2.12 Procedure for HNO_2_ Loading Contacts

Deionized water, a 10 M aqueous HNO_3_ stock solution, and an NaNO_2_ stock solution were combined in a 15 mL centrifuge tube to give a total of 8.5 mL of 20 mM HNO_2_ at the desired HNO_3_ concentration. A 1 mL portion of the aqueous phase was removed, transferred to a cuvette, and analyzed by UV–vis spectrophotometry. Immediately after NaNO_2_ addition, a 7.5 mL portion of 1.5 M DEHiBA that had been pre-equilibrated with the same concentration of HNO_3_ was added to the remaining 7.5 mL of aqueous HNO_2_ in HNO_3_. The sample was placed on a vortex mixer at 25 °C for 10 min and then centrifuged. UV–vis spectra of the aqueous and organic phases were recorded immediately after centrifuging. The total time elapsed between the NaNO_2_ addition and the measurement of the UV–vis spectra was generally 15–20 min. The distribution ratio for HNO_2_ (*D*_HNO2_) was calculated based on the ratio of the precontact and postcontact absorbances at 371 nm (i.e., as (Abs_initial_ – Abs_final_)/Abs_final_). The procedure was performed in triplicate at each HNO_3_ concentration. The reported error is the standard deviation for the 3 measurements.

For one of the three replicates, samples were prepared for Griess assays (*vide supra*) to confirm the mass balance of HNO_2_. Aliquots of the precontact and postcontact aqueous solutions were added to NaOH stocks that had been chilled on ice. The NaOH concentration was selected such that the final pH would be basic. For HNO_2_-loaded DEHiBA solutions, the organic phase was vortexed with an NaOH solution for 20 min at room temperature, then centrifuged. The aqueous phase was collected for Griess assays.

### 2.13 Procedure for U Oxide Dissolution and Reaction Stoichiometry Analysis

A 2 mL cryo-vial was charged with 35 mg of solid U oxide and 1.8 mL of 1.5 M DEHiBA in *n*-dodecane that had been precontacted twice with 6 M HNO_3_ ([HNO_3_]_org_ = 1.3 M). The resulting suspension was placed on a shaker plate at ambient temperature for 45 min. The vial was then centrifuged to promote phase disengagement and the organic phase was collected and analyzed. A sample for Griess analysis was prepared immediately after the organic phase was collected by adding a 0.1 mL aliquot of the organic phase to 1 mL of 0.35 M NaOH and shaking for 20 min; analysis of the resulting aqueous phase was performed as described in Section 2.11. A UV–vis spectrum of the U-loaded organic phase was recorded. Aliquots of the organic phase were added to 0.2 M ammonium oxalate and potentiometric titrations were performed. ICP-OES samples were prepared by adding 0.1 mL of organic phase to 10 mL of 5% HNO_3_. The samples analyzed for organic-phase water concentration (Figure S8 and Figure S9) were prepared on a larger scale (approximately 100 mg U oxide in 5 mL 1.5 M DEHiBA) and were mixed by adding a PTFE-coated stir bar to the vial and stirring for 90 min. The uncertainties in the reaction stoichiometries are based on propagation of the uncertainties in the underlying measurements.

### 2.14 Procedure for U Oxide Dissolution at High U Loading

A 10 mL polypropylene cryo-vial or an 8 mL glass tube was charged with a PTFE-coated stir bar, 480 mg of ε-UO_3_ or 470 mg of α-U_3_O_8_, and 4 mL of 1.5 M DEHiBA that had been precontacted twice with 6 M HNO_3_. After stirring for 3 h, the sample was centrifuged and the organic phase was decanted and analyzed. A sample for Griess analysis was prepared by adding 0.1 mL of organic phase to 5 mL of 0.05 M NaOH and placing on a shaker plate for 20 min; analysis of the resulting aqueous phase was performed as described in Section 2.11. A sample for UV–vis was prepared by adding a 0.2 mL aliquot of the organic phase to 0.6 mL of HNO_3_-loaded 1.5 M DEHiBA. ICP-OES samples were prepared by adding 0.1 mL of organic phase to 40 mL of 5% HNO_3_. Potentiometric titrations were performed on 0.2 mL aliquots of organic phase added to 25 mL of 0.2 M ammonium oxalate. The uncertainties in the reaction stoichiometries are based on propagation of the uncertainties in the underlying measurements. The volume of aqueous phase was estimated by using an adjustable volume micropipette to collect as much of the aqueous phase as possible.

## 3 Results and Discussion

### 3.1 Preparation and Characterization of Uranium Oxides

**Figure 2:**
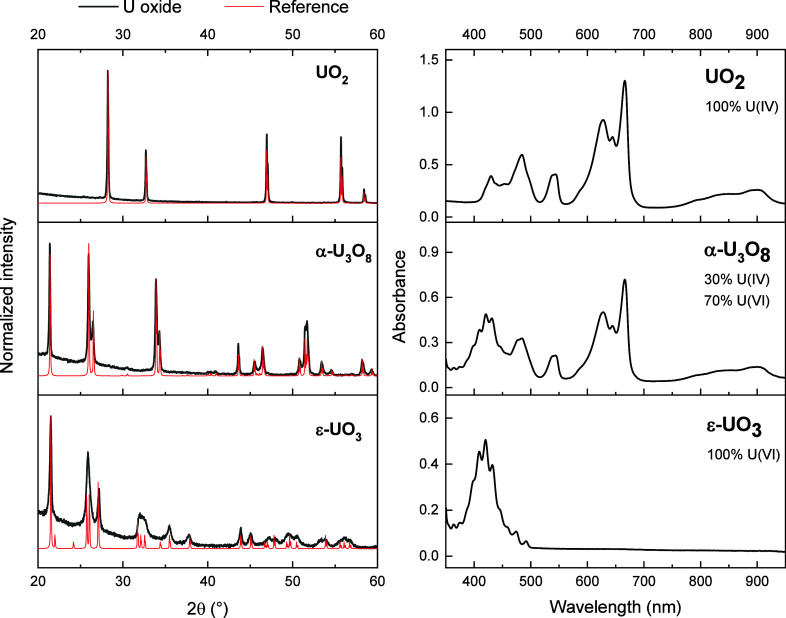
(Left) PXRD data for synthesized U oxides (black) compared to reference data (red) for UO_2_ (PDF 01–071–6146), α-U_3_O_8_ (PDF 04–007–1246), and ε-UO_3_ (PDF 00–018–1429). (Right) UV–vis spectra of UO_2_, α-U_3_O_8_, and ε-UO_3_ after digestion in 85% H_3_PO_4_ containing 0.141 M Na_2_SO_4_.

The U oxides used for the studies described in this work were synthesized starting from UO_2_ powder. As discussed in the Introduction, current research efforts in the US are focused on NO_2_ voloxidation. However, an alternative synthetic procedure was employed to generate the U oxides used for the studies described in this work to avoid the use of highly toxic NO_2_ gas. Oxidation of UO_2_ under O_2_ at 440 °C resulted in a color change from dark brown to black, indicating formation of α-U_3_O_8_. Following a literature procedure,^31^ further oxidation of the α-U_3_O_8_ in a mixture of O_3_ and O_2_ generated ε-UO_3_ as a bright orange powder. Photos of the three U oxides are shown in Figure S2 and PXRD data confirming the phases of the oxides are shown in Figure 2.

Evidence for the identity and purity of the U oxides was obtained by UV–vis analysis of samples digested in phosphoric acid (H_3_PO_4_). Dissolution of uranium oxides of various stoichiometry in concentrated phosphoric acid was proposed four decades ago as a useful method to determine the oxygen-to-uranium ratio in these materials^39,40^ and the applicability of this technique to determination of the oxidation state of U metal corrosion products in Hanford K-basin sludge was demonstrated in 2007–2010.^41^ In this method, samples are digested in 85% H_3_PO_4_ containing 20 g/L Na_2_SO_4_ which is a complexing but nonoxidizing medium that preserves the net oxidation state of U. As shown in Figure 2, the UV–vis spectrum of the digested UO_2_ showed only peaks belonging to U(IV) with no evidence for U(VI). Similarly, the UV–vis spectrum of the digested ε-UO_3_ confirmed full conversion to U(VI). The spectrum of α-U_3_O_8_ contained an approximately 2:1 ratio of U(VI):U(IV) consistent with U_3_O_8_.

Further confirmation of the identity of the ε-UO_3_ was obtained from the Raman spectrum of this material, which shows a major peak at 653 cm^–1^ and two minor doublets at approximately 500 and 800 cm^–1^ (Figure S3) consistent with spectral data reported in the literature.^31^ In addition, U L_3_-edge XANES data were collected with a laboratory scale XAS spectrometer. The edge positions, determined as maxima of the first derivative peak, are 17168.0 eV for UO_2_, 17170.2 eV for U_3_O_8_, and 17170.8 eV for ε-UO_3_ (Figure S4). The shift of the edge position to higher energy along this series is indicative of an increase in formal U oxidation state, and the magnitude of the differences in the edge positions between the U oxides are consistent with values reported in the literature.^37,38^

### 3.2 Characterization of HNO_2_ in DEHiBA

To enable quantification of HNO_2_, which is expected to be one of the products formed during dissolution of α-U_3_O_8_ and UO_2_ (reactions 3 and 4), its UV–vis spectroscopic signature in 1.5 M DEHiBA was determined at multiple HNO_3_ concentrations. To ensure that the organic phase HNO_3_ concentration would be at equilibrium, the organic phases used in these experiments were first pre-equilibrated twice with an equal volume of 1–6 M aqueous HNO_3_. The resulting organic phase was then contacted with an aqueous solution containing 20 mM HNO_2_ at the same HNO_3_ concentration used in the precontact. The HNO_2_ loading contacts were performed in vessels with minimal headspace and UV–vis spectra were recorded as quickly as possible after the contact was performed (approximately 15 to 20 min after combining the aqueous and organic phases) to avoid significant HNO_2_ decomposition due to NO_*x*_ production and off-gassing. The postcontact aqueous HNO_2_ concentrations were determined by comparing the precontact and postcontact UV–vis spectra, and the balance of the HNO_2_ was assumed to be in the organic phase. To confirm that this assumption is valid, at least one set of samples at each HNO_3_ concentration was also analyzed using a colorimetric test for nitrite (Griess assay^36^). We note that under the conditions used to strip the HNO_2_ out of the organic phase, NO_*x*_ species other than HNO_2_ could also be detected by the Griess assay. In particular, NO_2_ reacts with NaOH to generate nitrite:
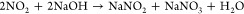
6

However, the HNO_2_ concentrations determined from the Griess assays were consistent with those determined based on the UV–vis spectra of the aqueous phases (Figure S5). Furthermore, the HNO_2_ recoveries after the contact were close to 100% (Figure S5). Taken together, these results suggest that there is not a significant amount of NO_2_ in the organic phase.

**Figure 3:**
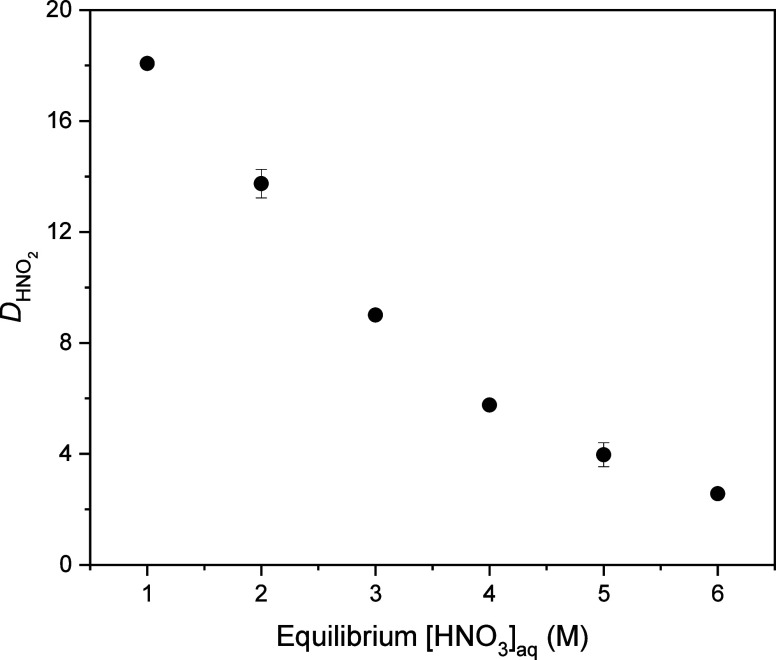
Distribution ratio of HNO_2_ as a function of equilibrium aqueous HNO_3_ concentration for extraction by 1.5 M DEHiBA. Error bars are plotted as one standard deviation for all data points, including where they are not visible in the figure.

As shown in Figure 3, HNO_2_ is strongly extracted by 1.5 M DEHiBA at low acidity. The decrease in the HNO_2_ distribution ratio (*D*_HNO2_) with increasing HNO_3_ concentration can be attributed to the decreased amount of free DEHiBA available for HNO_2_ binding at high acidity.^23^ This phenomenon has also been observed for extraction of HNO_2_ by 30% TBP in *n*-dodecane^42,43^ and for extraction of HNO_2_ by 1 M solutions of DEHiBA and other monoamides.^44^

**Figure 4:**
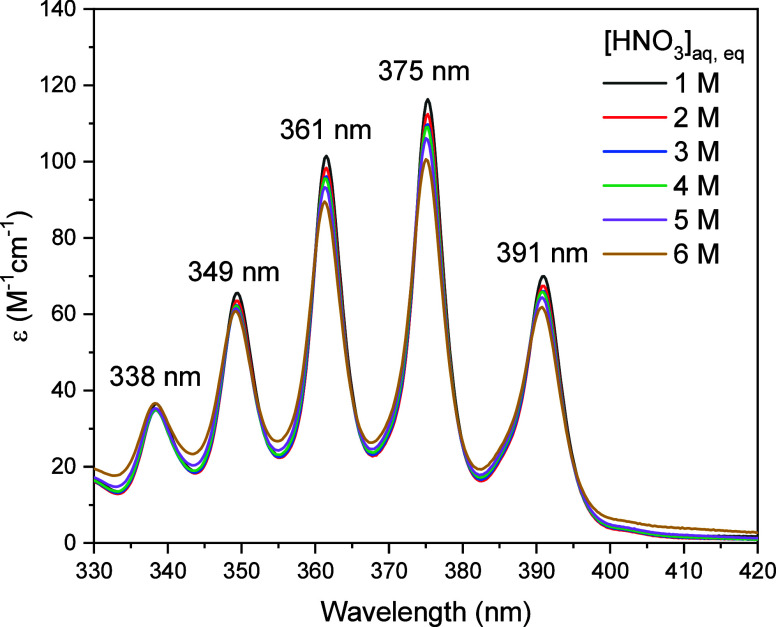
UV–visible spectra of HNO_2_ in 1.5 M DEHiBA at different equilibrium aqueous-phase acidities.

The UV–vis spectra of the postcontact organic phases (Figure 4) contain a series of relatively sharp peaks with molar absorptivities in the 50–120 M^–1^cm^–1^ range (Table S1). Peaks with similar λ_max_ and ε have been observed for HNO_2_ in TBP^43^ and in 1 M solutions of various monoamides including DEHiBA^44^ as well as in other organic solvents^45^ and in the gas phase.^46^ The features have been assigned to a vibronic progression of the nonbonding to antibonding transition of a mixture of *cis*- and *trans*-HNO_2_.^46^ There is a modest decrease in molar absorptivity with increasing acidity for the peaks at 349, 361, 375, and 391 nm (Figure S6). This phenomenon (referred to as “quenching”) has been observed previously for HNO_2_ in TBP^43^ and was attributed to decomposition of HNO_2_ via the equilibrium:
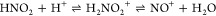
7based on the behavior of HNO_2_ in strongly acidic aqueous media. However, the nature and reactivity of the species formed by interaction of HNO_2_ with HNO_3_ are likely to be different in aqueous media compared to a more nonpolar organic phase. We hypothesize that the changes in the UV–vis spectrum are due to hydrogen bonding of HNO_2_ to HNO_3_ and/or formation of (DEHiBA)_*x*_•(HNO_2_)_*y*_ complexes with different stoichiometries at different acidities.

As reported by Gogolski,^44^ over time the characteristic peaks of HNO_2_ decrease in intensity and several new features appear, particularly for samples prepared at high acidity (Figure S7). A detailed study of this phenomenon is beyond the scope of this work. However, there is no evidence for the presence of this species in the UV–vis spectra from direct extraction experiments (*vide infra*), which suggests that it either does not form under the conditions in the direct extraction experiments or that in the presence of U it forms a species with a similar spectrum to that of UO_2_(NO_3_)_2_ in DEHiBA.

### 3.3 Water Extraction by 1.5 M DEHiBA

**Figure 5:**
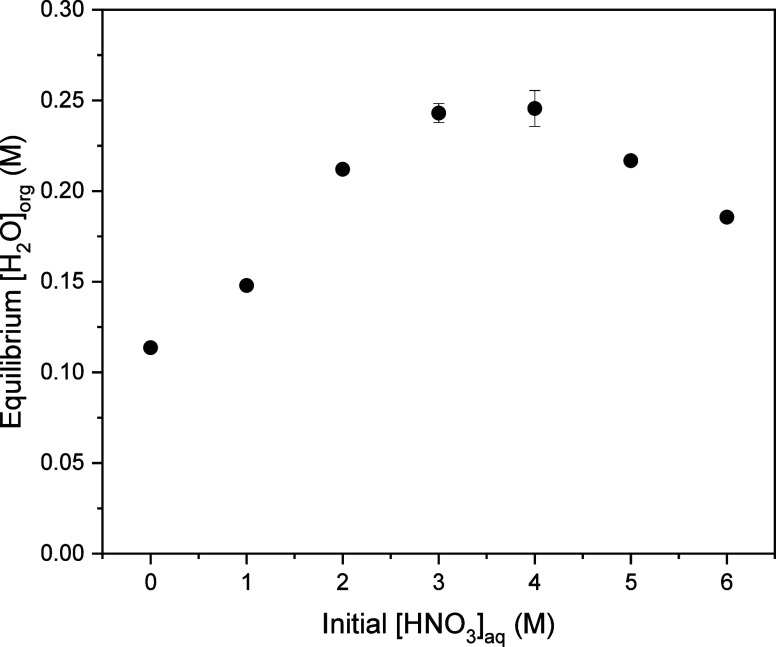
Equilibrium organic-phase H_2_O concentration as a function of initial aqueous-phase acidity for 1.5 M DEHiBA. Error bars are plotted as the difference between two replicate analyses, including where they are not visible in the figure.

Karl Fischer titrations were performed to determine the equilibrium water concentrations in HNO_3_-loaded 1.5 M DEHiBA. As shown in Figure 5, the equilibrium organic-phase H_2_O concentration first increases and then decreases with increasing HNO_3_ concentration, with the maximum H_2_O content observed for initial aqueous-phase HNO_3_ concentrations of 3–4 M. Similar behavior was observed for extraction of H_2_O by 1 M DEHiBA in hydrogenated tetrapropylene (TPH).^47^ This behavior can potentially be attributed to differences in the mechanism of H_2_O extraction for the three major species in these solutions (DEHiBA, HNO_3_•(DEHiBA)_2_, and HNO_3_•DEHiBA).^23^ The differences in the relative populations of these species at different acidities then result in coextraction of different amounts of water. In support of this hypothesis, the highest H_2_O concentrations are observed under conditions where the concentration of HNO_3_•(DEHiBA)_2_ is highest.^23^

### 3.4 Determination of Reaction Stoichiometries for U Oxide Dissolution

Initial studies of the dissolution of UO_2_, α-U_3_O_8_, and ε-UO_3_ were performed with a target loading of 0.07 M U in order to allow direct spectroscopic measurement of the loaded organic phase. These conditions should also result in relatively low HNO_2_ concentrations which disfavor HNO_2_ disproportionation. To further disfavor off-gassing of NO_*x*_ species, the reactions were performed in vials with minimal headspace and were analyzed after mixing for a relatively short period of time (45 min).

When HNO_3_-loaded DEHiBA was added to ε-UO_3_, the yellow color characteristic of uranyl nitrate was evident within a few minutes of mixing at ambient temperature. In contrast, a large amount of undissolved solid persisted for at least 20 min when α-U_3_O_8_ and UO_2_ were dissolved under the same conditions. This was followed by rapid dissolution of the majority of the solid over the course of approximately 5 min. We hypothesize that this behavior is due to autocatalytic production of NO_*x*_ during dissolution, which has been suggested previously for dissolution of UO_2_ in aqueous HNO_3_.^48^ However, further investigation would be needed to test this hypothesis.

Even in these small-scale, low U-loading experiments, a separate aqueous phase was evident after the samples were centrifuged (Figure S8). Karl Fischer titrations of the organic phase before and after dissolution showed no significant increase in water concentration following U dissolution (Figure S9) indicating that the production of water by the dissolution (eqs 3-5) results in formation of a separate aqueous phase. Due to the small scale of these experiments, it was not practical to quantify the volume of the aqueous phase produced during the reaction or analyze its composition. Assuming the stoichiometries in eqs 3-5 are correct, the reaction will produce one molar equivalent of water per uranium dissolved, which would result in an organic to aqueous phase volume (O:A) ratio of approximately 800:1 for the experiments described here. Based on the distribution ratios for HNO_2_ reported in this work and previously reported distribution ratios for HNO_3_ and U in 1.5 M DEHiBA,^23^ the amount of HNO_3_, HNO_2_, and U that is expected to strip into the aqueous phase under these conditions is small (i.e., within analytical uncertainty).

**Figure 6:**
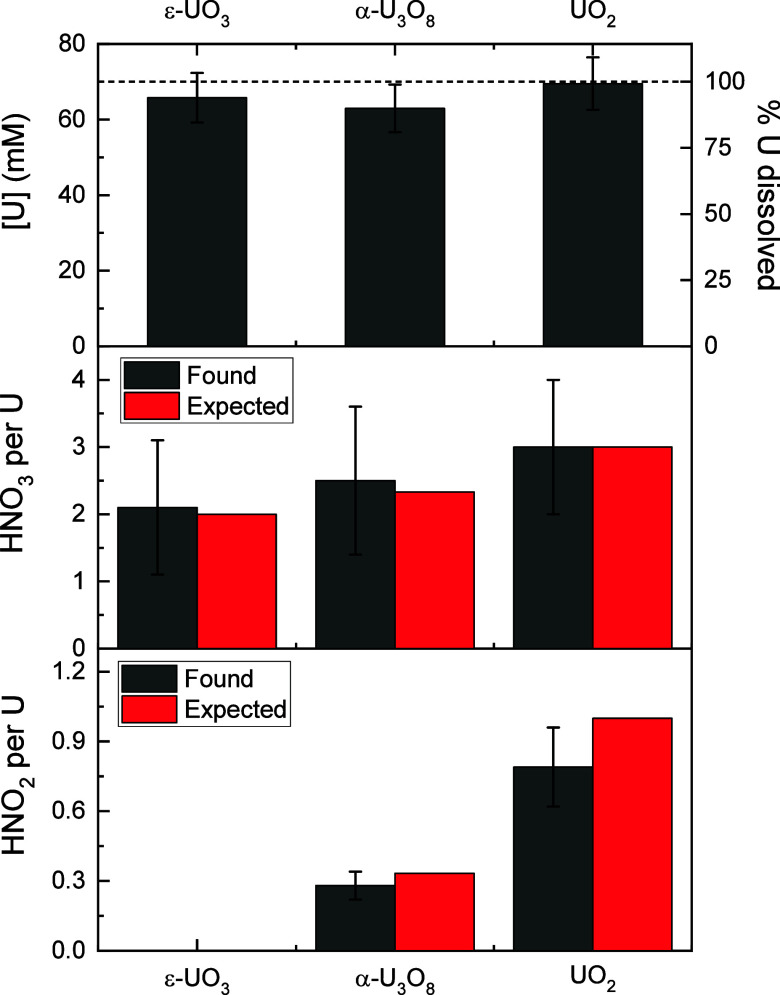
(Top) Comparison of the organic-phase U concentrations from dissolution of ε-UO_3_, α-U_3_O_8_, and UO_2_ in 1.5 M DEHiBA determined by ICP-OES. The theoretical U concentration (assuming 100% dissolution and no volume change upon dissolution) is shown as a dashed line. (Middle) Comparison of observed reaction stoichiometries for HNO_3_ consumption to the expected values based on eqs 3–5. (Bottom) Comparison of the observed reaction stoichiometries for HNO_2_ production to the expected values based on eqs 3–5.

**Figure 7:**
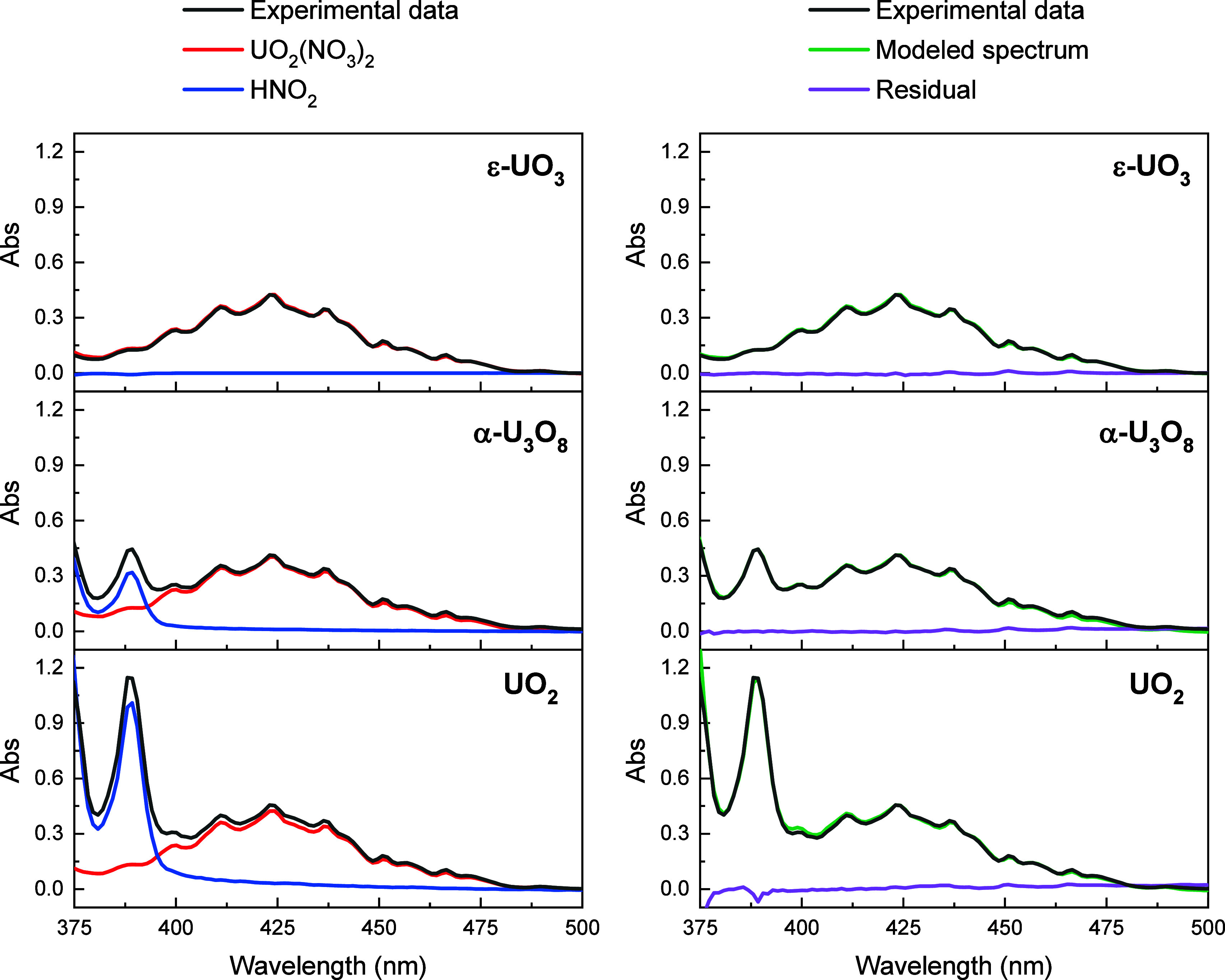
UV–vis spectra of the loaded organic phase obtained from dissolution of ε-UO_3_ (top), α-U_3_O_8_ (middle), or UO_2_ (bottom) in 1.5 M DEHiBA with a target U loading of 0.07 M. The experimental data is shown in black. In the left panel, the contributions from uranyl nitrate and HNO_2_ are shown in red and blue, respectively. On the right, the modeled spectrum is shown in green and the residual (experimental – modeled) is shown in purple.

ICP-OES analysis of the U-loaded organic phase indicated greater than 90% dissolution after 45 min with only minor differences in the extent of dissolution for the 3 oxides (Figure 6). Representative UV–vis spectra of the organic phase after dissolution of the uranium oxides are shown in Figure 7, along with deconvolution of the HNO_2_ and uranyl nitrate signals. There is no HNO_2_ formed during dissolution of ε-UO_3_, and dissolution of UO_2_ produced approximately 3x as much HNO_2_ as dissolution of α-U_3_O_8_. The HNO_2_ concentrations determined via Griess assay were within experimental error of the concentrations determined from the UV–vis spectra. As shown in Figure 6, dissolutions of α-U_3_O_8_ and UO_2_ produce 0.28 and 0.79 equiv of HNO_2_ per U, respectively. These are moderately lower than expected based on eqs 3 – 5 which could be due to partial decomposition of HNO_2_ over the course of the experiment and/or production and off-gassing of other NO_*x*_ species. Regardless, these results demonstrate that a significant amount of the NO_*x*_ produced by dissolution of UO_2_ and α-U_3_O_8_ remains in solution as HNO_2_ under these conditions.

The free acid concentrations after U dissolution were determined by potentiometric titration of aliquots of the organic phase in 0.2 M ammonium oxalate. For the ε-UO_3_ dissolution in which no HNO_2_ is present, the stoichiometry with respect to HNO_3_ consumption can be determined directly from the change in free acid concentration before and after U dissolution. For the α-U_3_O_8_ and UO_2_ dissolutions, the stoichiometry can be determined after correcting for the contribution of HNO_2_ to the total free acidity in the sample. Although the uncertainties are large due to the small (approximately 0.14 M) change in free acid concentration in these experiments, the observed stoichiometries of 2.1, 2.5, and 3.0 equiv of HNO_3_ consumed per U for ε-UO_3_, α-U_3_O_8_, and UO_2_, respectively, are within experimental error of the hypothetical stoichiometries in reactions 3 - 5.

### 3.5 Dissolution of U Oxides at High U Loading

Since α-U_3_O_8_ and ε-UO_3_ are expected to be the primary products of voloxidation, further direct extraction studies were performed with these U oxides at a higher loading that could form the basis of a U recovery flowsheet. Specifically, a target U loading of 100 g/L (0.42 M) was selected based on a previous study with 1.5 M DEHiBA in Isane, which indicated that above this U loading, viscosity increases rapidly with increasing U concentration.^49^

**Figure 8:**
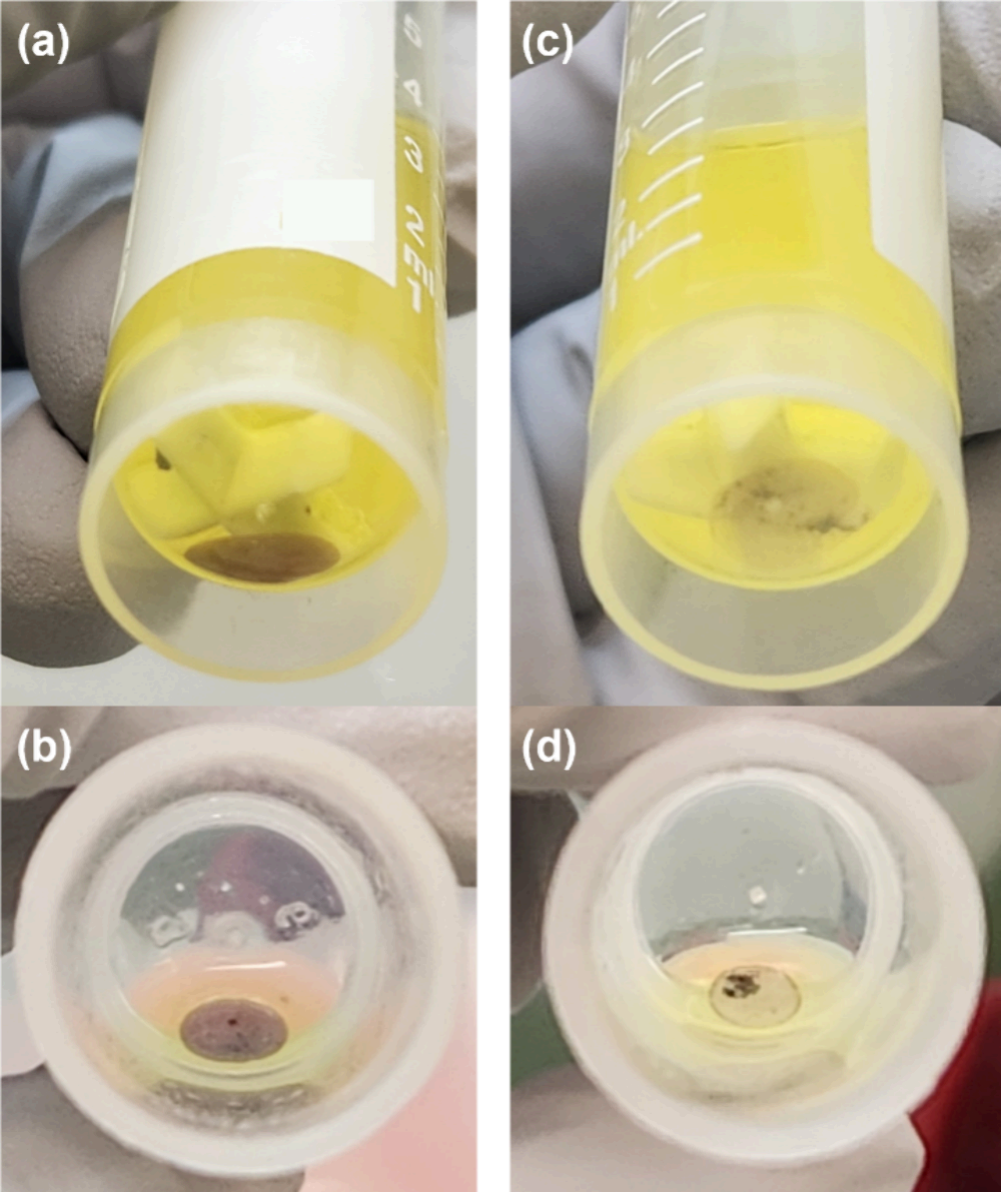
Photos of the aqueous phases formed upon dissolution of ε-UO_3_ (a, b) or α-U_3_O_8_ (c, d) in 1.5 M DEHiBA. The photos in the top row show the bottom of the reaction vessel immediately after centrifuging, and the photos in the bottom row were taken from the top of the vessel after removing the stir bar and decanting the majority of the organic phase. The target U concentration in these experiments was 0.42 M.

Within a few minutes of stirring ε-UO_3_ with HNO_3_-loaded DEHiBA in a polypropylene vial at ambient temperature, a yellow solution was produced. The undissolved solids clumped together at the beginning of the reaction (Figure S10) but after 20 min very little undissolved solid remained. After centrifuging, the aqueous phase formed a single droplet and a small amount of brown solid gathered in and around the aqueous droplet (Figure 8). The aqueous phase volume was approximately 40 μL corresponding to an organic to aqueous ratio of 100:1. This is slightly larger than the expected aqueous phase volume of 30 μL; however, it is difficult to precisely determine the droplet volume due to the relatively small scale of the reaction and the presence of entrained solids. When the reaction was conducted on the same scale in a glass tube, the solids adhered to the walls of the vial but the majority of the material still dissolved over the course of several hours (Figure S12). Additionally, after centrifuging the aqueous phase was comprised of multiple small droplets adhered to the glass rather than a discrete single droplet. The qualitative differences in the behavior of the dissolution in glass and polypropylene vials are likely caused by the difference in the polarity of the surfaces of the container. Glass vials have a polar, protic surface which the U oxides and water adhere to whereas the polyethylene vials have a nonpolar, aprotic surface, so the U oxides adhere to the polar, protic aqueous phase rather than to the vial. This observation demonstrates that it will be important to consider the physical properties of undissolved solids and their interaction with both water and the dissolver vessel when designing equipment for use in direct extraction. In both reactions, near-quantitative dissolution of U was achieved (average [U] = 0.42 ± 0.06 M) after stirring for 3 h at ambient temperature. No HNO_2_ was produced in either case and the HNO_3_ consumption was the same within error for the two experiments. The resulting stoichiometry of 2.1 ± 0.3 equiv of HNO_3_ consumed per equivalent of U dissolved agrees well with both eq 5 and the results obtained at lower U loading.

Qualitatively, the dissolution of α-U_3_O_8_ was moderately slower than the dissolution of ε-UO_3_. The reaction mixture remained a black slurry for the first 10 min, at which point the undissolved solids clumped together, revealing a yellow solution (Figure S11). The remaining undissolved solid became suspended in the aqueous phase before dissolving almost entirely. After the dissolution was stopped, a droplet of aqueous phase was again evident and contained only a small amount of black undissolved solid (Figure 8). When the dissolution was conducted in a glass vial the solids again adhered to the walls of the vial and dissolved more slowly (Figure S12). However, in both cases ICP-OES analysis of the organic phase after stirring for 3 h indicated U concentrations within experimental error of the target concentration of 0.42 M U (Figure S13).

The UV–vis spectra of diluted aliquots of the organic phase taken after the reaction had stirred for 3 h indicated somewhat different HNO_2_ concentrations for the dissolutions conducted in glass and polypropylene vials. The reason for this difference in behavior is unclear. One possible explanation is related to the difference in dissolution rate; since the dissolution in the polypropylene vial was faster, the HNO_2_ was in solution for longer which would result in a greater extent of decomposition. Furthermore, there may have been high local HNO_2_ concentrations produced during the more rapid dissolution process which could lead to HNO_2_ disproportionation. In addition, the diameter of the glass vial was narrower which resulted in a visible concentration gradient within the vial, particularly at early time points (Figure S12), and suggests the HNO_2_ concentration at the liquid–gas interface may have been low, preventing NO_*x*_ off-gassing. For both reactions the Griess assays indicated higher HNO_2_ concentrations than the UV–vis spectra of diluted samples (Figure S13) which suggests that there is a change in NO_*x*_ speciation upon dilution of the sample and/or that both HNO_2_ and other NO_*x*_ species that react with NaOH to produce nitrite (most likely NO_2_) are present in the organic phase. The exact stoichiometry with respect to HNO_2_ production is therefore unclear, but is between 0.20 and 0.35 equiv of HNO_2_ per U in reasonable agreement with eq 4. The data again demonstrate that a significant amount of NO_*x*_ produced by α-U_3_O_8_ dissolution remains in the organic phase as HNO_2_ under these conditions. However, NO_*x*_ speciation is complex and will likely change under different conditions, particularly for UNF where additional HNO_2_ will be produced by HNO_3_ radiolysis.

The uncertainty in HNO_2_ concentration also results in uncertainty in the post-dissolution HNO_3_ concentration since the titrated free acid concentration after U dissolution is the sum of the HNO_2_ and HNO_3_ concentrations. If the stoichiometry in eq 4 were followed (i.e., if HNO_2_ were the only NO_*x*_ product from dissolution), the net change in free acid concentration would be two equivalents per U. The average observed change across the two experiments is 2.3 ± 0.4 equiv of H^+^ per U, which implies that at least some HNO_3_ is converted to NO_*x*_ species other than HNO_2_. Accounting for the 0.20–0.35 equiv of HNO_2_ produced per U, approximately 2.5 equiv of HNO_3_ are consumed per equivalent of U dissolved (Figure S13), which agrees with the stoichiometry determined at lower U loading.

## 4 Conclusions

Process intensification where industrial process steps are combined has the potential to change the economics of nuclear reprocessing by reducing the plant footprint and the generation of secondary waste. In this work, the concept of direct extraction has been coupled to the uranium-selective extractant DEHiBA at an increased extractant concentration of 1.5 M. The generation of a loaded organic phase containing U concentrations suitable for further processing has been demonstrated. To better understand the challenges that will be faced during process scale-up, the stoichiometries of the reactions for dissolving UO_2_, α-U_3_O_8_, and ε-UO_3_ have been examined. The results suggest that the reactions follow eqs 3-5, although particularly with respect to NO_*x*_ generation there may be additional considerations when transitioning to irradiated fuel. The H_2_O generated by the dissolution results in formation of a separate aqueous phase which will need to be taken into account during flowsheet design, as will the treatment of any undissolved solids. Maturing this technology will also require investigating the behavior of other constituents of UNF under direct extraction conditions. Ultimately, the direct extraction of U from voloxidzed UNF into DEHiBA appears to be a promising approach if the extraction can be performed with real irradiated fuel under conditions that do not result in excessive radiolytic solvent degradation.

## Supporting Information Available

Additional characterization data for solid uranium oxides, comparison of HNO_2_ distribution ratios determined from Griess assays and UV–vis spectra, molar absorptivities of HNO_2_ in 1.5 M DEHiBA at different acidities, photos of uranium oxide dissolutions, and additional characterization of uranium-loaded organic phases (PDF)
