# Supplementary material for: Direct Extraction of Uranium Oxides with N,N-Di(2-ethylhexyl)isobutyramide
Source: ACS Omega. 2025 Mar 28;10(13):13674–84. doi: 10.1021/acsomega.5c01103 (PMC11983218; doi:10.1021/acsomega.5c01103)
Supplement: Supplementary file 1 — ao5c01103_si_001.pdf [file ao5c01103_si_001.pdf]

Supporting Information for

Direct Extraction of Uranium Oxides with DEHiBA

Amy L. Speelman, Daria Boglaienko, Avalon B. Tarbet-Mendoza, Nathan P. Bessen, Ashley N. Williams, Sergey I. Sinkov, Bruce K. McNamara, Gregg J. Lumetta, Gabriel B. Hall**

Pacific Northwest National Laboratory, Richland, Washington 99352, USA

Table of Contents

1. Supplementary data for Section 3.1 (preparation and characterization of uranium oxides)........2
2. Supplementary data for Section 3.2 (characterization of HNO_2 in DEHiBA)............................4
3. Supplementary data for Section 3.4 (determination of reaction stoichiometries for U oxide dissolution)..7
4. Supplementary data for Section 3.5 (dissolution of U oxides at high U loading) 8

1. Supplementary data for Section 3.1 (preparation and characterization of uranium oxides)

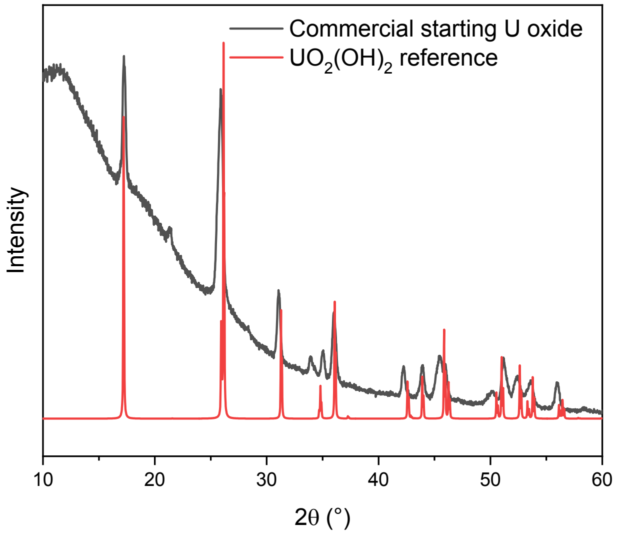

Figure S1. PXRD of the commercial U_3O_8 used as the starting material for synthesis of U oxides compared to reference data for $UO_2(OH)_2$.

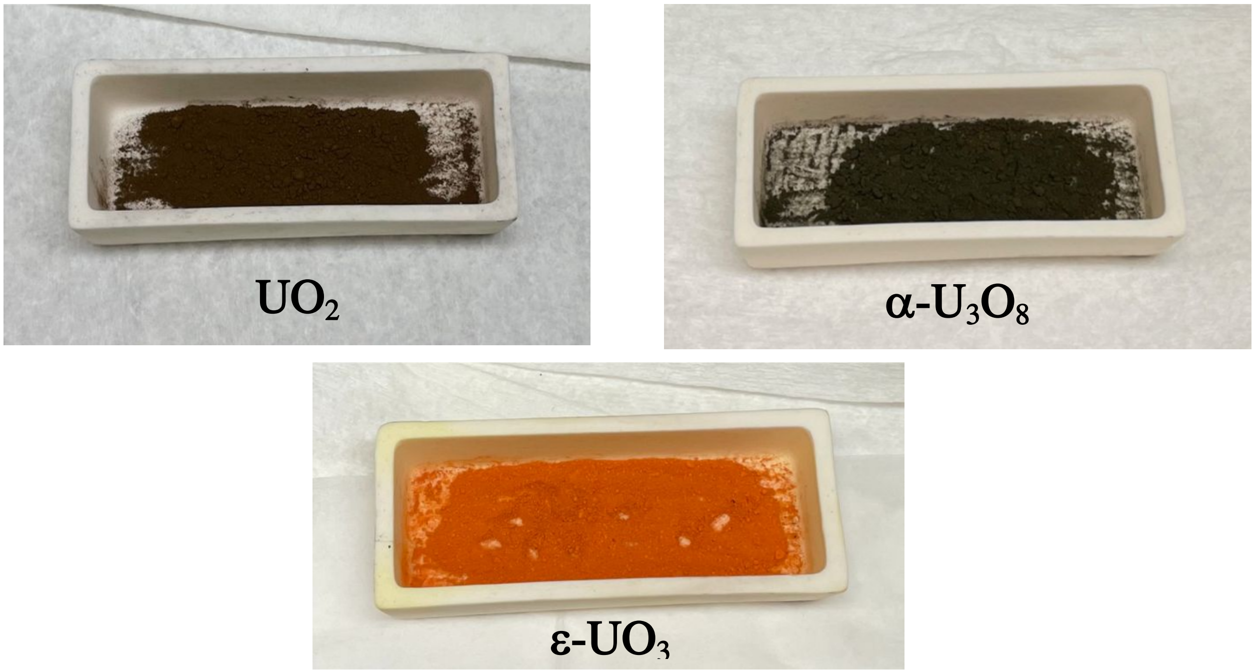

Figure S2. Photos of uranium oxides used for the studies described in this work.

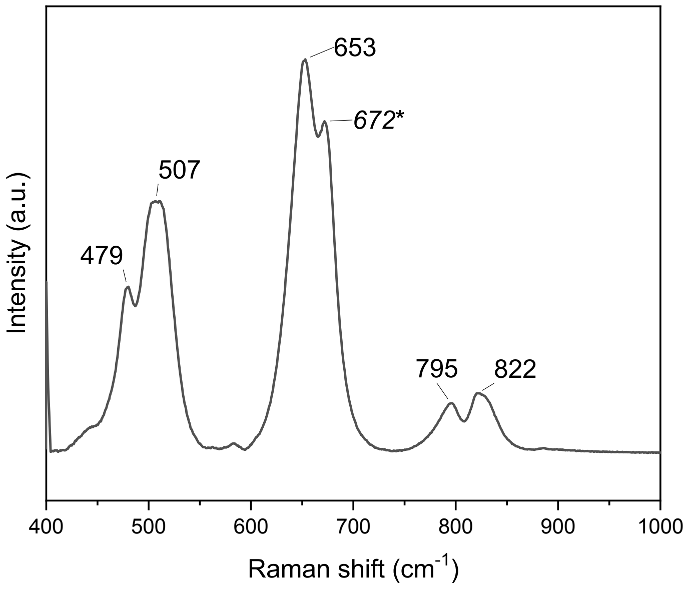

Figure S3. Raman spectrum of ε-UO_3 collected using a 670.39 nm excitation wavelength. The peak at 672 cm^{-1} marked with an asterisk is assigned to a magnetite (Fe_3O_4) impurity[1], likely arising from corrosion in the apparatus used for the synthesis of this material.

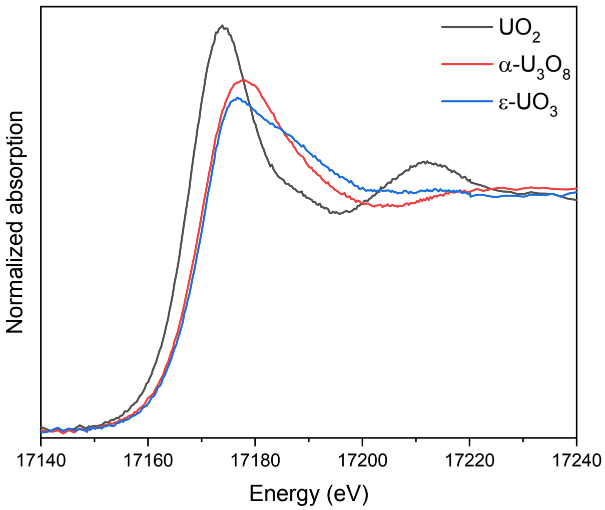

Figure S4. U L_3-edge XANES for UO_2 (black), α-U_3O_8 (red), and ε-UO_3 (blue).

---

[1] Gasparov, L. V.; Tanner, D. B.; Romero, D. B.; Berger, H.; Margaritondo, G.; Forró, L. Infrared and Raman studies of the Verwey transition in magnetite. *Phys. Rev. B* **2000**, *62*, 7939-7944. DOI: 10.1103/PhysRevB.62.7939.

2. Supplementary data for Section 3.2 (characterization of HNO_2 in DEHiBA)

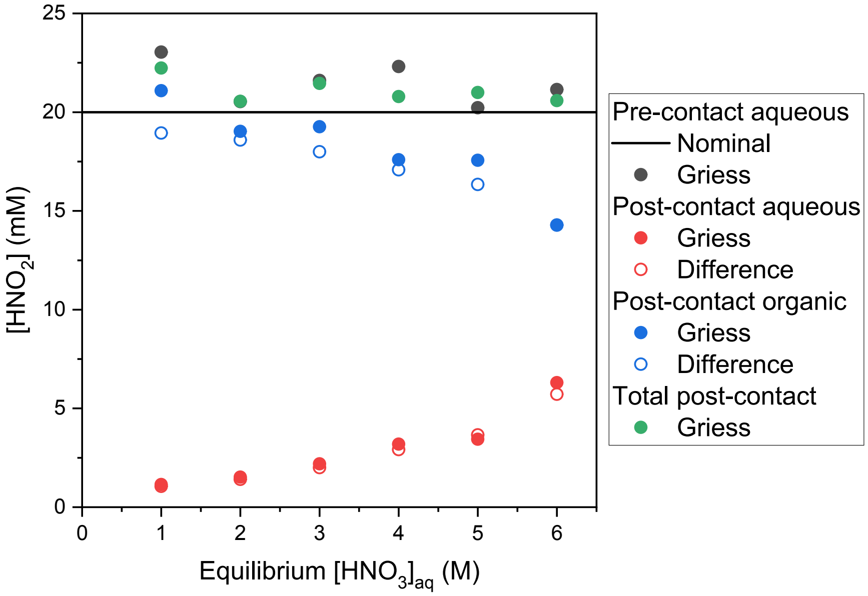

Figure S5. HNO_2 concentrations in aqueous and organic phases from contact of 20 mM HNO_2 at varying equilibrium aqueous phase HNO_3 concentration with 1.5 M DEHiBA. The results from Griess assays are shown as solid circles, and the corresponding concentrations determined based on the difference in absorbance in the aqueous phase before and after the contact are shown as open circles. The nominal total HNO_2 concentration (20 mM) is shown as a solid line. The sum of the HNO_2 in the post-contact aqueous and organic phases determined by the Griess assay (green circles) is within 10% of the amount of HNO_2 in the pre-contact aqueous phase (black circles) at all HNO_3 concentrations.

Table S1. Distribution ratios and molar absorptivities at varying wavelengths for HNO_2 in 1.5 M DEHiBA in *n*-dodecane at varying acidity.

			ε ($M^{-1}cm^{-1}$)			
$[HNO_3]_{aq, eq}$ (M)	$[HNO_3]_{org, eq}$ (M)[a]	D_{HNO2}	349 nm	361 nm	375 nm	391 nm
1	0.13	18.1	64	98	115	70
2	0.42	13.7	63	96	112	67
3	0.71	9.0	62	95	110	66
4	0.97	5.8	62	93	108	65
5	1.20	4.0	60	90	104	62
6	1.34	2.6	59	87	99	60

[a] The organic phase acidity was determined by potentiometric titration of the HNO_3-loaded organic phase before performing the HNO_2 contact.

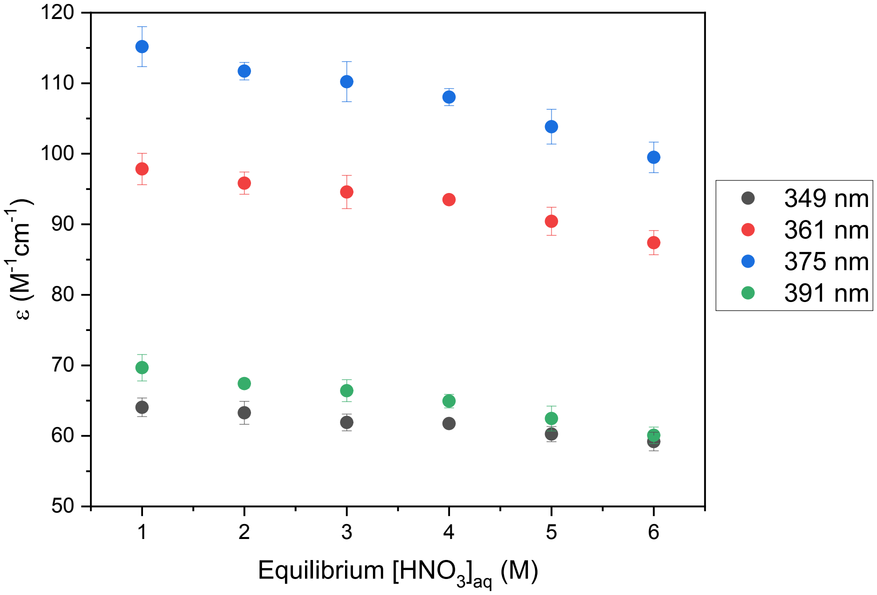

Figure S6. Organic-phase molar absorptivity for HNO_2 as a function of equilibrium aqueous-phase HNO_3 concentration at different wavelengths.

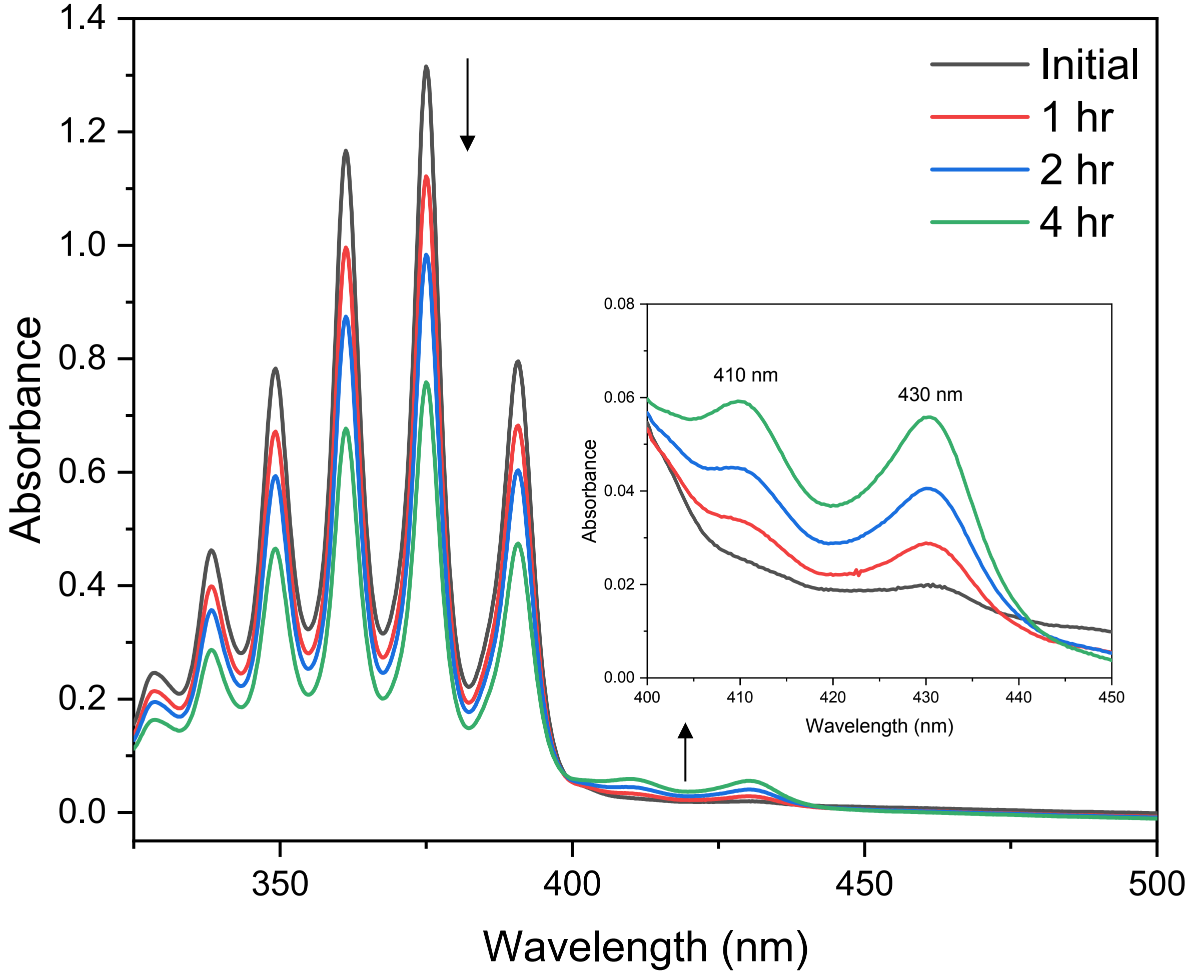

Figure S7. UV-vis spectra showing the decomposition of HNO_2 in 1.5 M DEHiBA containing approximately 1.3 M HNO_3 during stirring of 4 mL of sample in an 8 mL vial. The inset shows the appearance of new features at 410 nm and 430 nm. Based on Griess assays, the initial HNO_2 concentration was 24 mM, and the HNO_2 concentration after 4 hours was 13 mM.

3. Supplementary data for Section 3.4 (determination of reaction stoichiometries for U oxide dissolution)

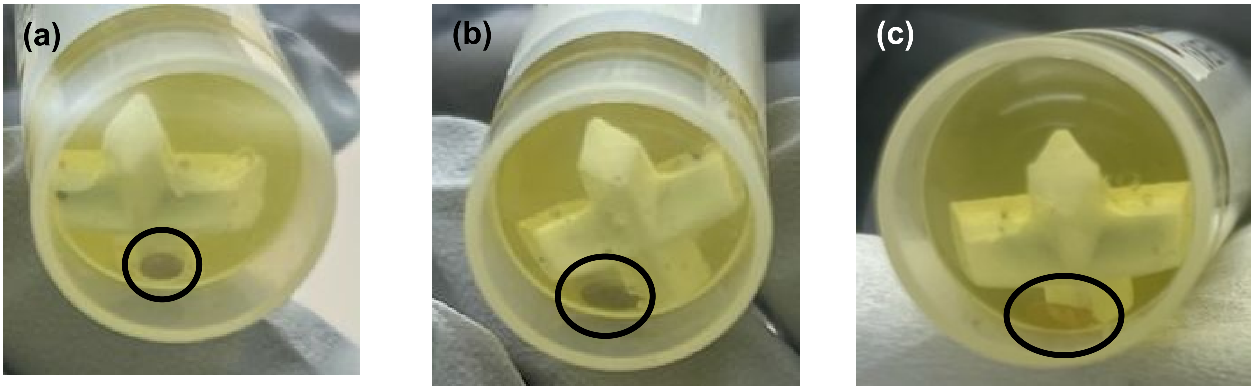

Figure S8. Photos showing the droplet of aqueous phase formed after stirring 100 mg ε-UO_3 (a), α-U_3O_8 (b), or UO_2 (c) in 5 mL of 1.5 M DEHiBA for 1.5 hours at ambient temperature.

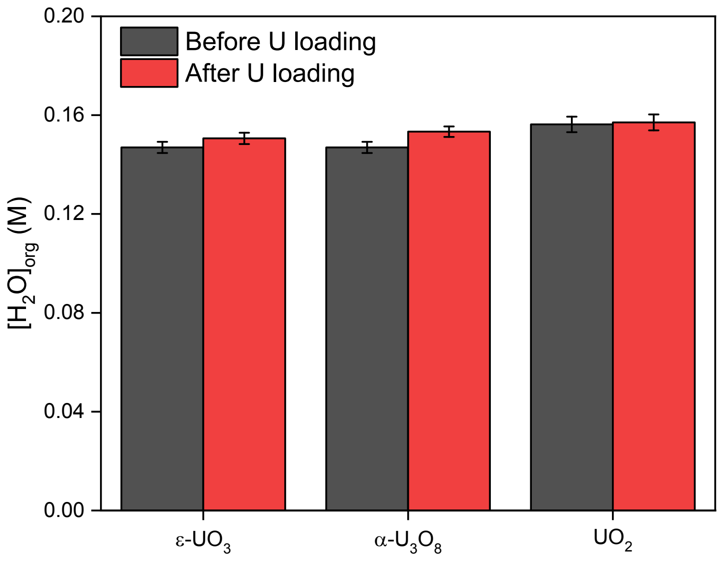

Figure S9. Organic-phase water concentrations before and after U loading for dissolution of 100 mg U oxide in 5 mL of 1.5 M DEHiBA (target [U] = 0.07 M).

4. Supplementary data for Section 3.5 (dissolution of U oxides at high U loading)

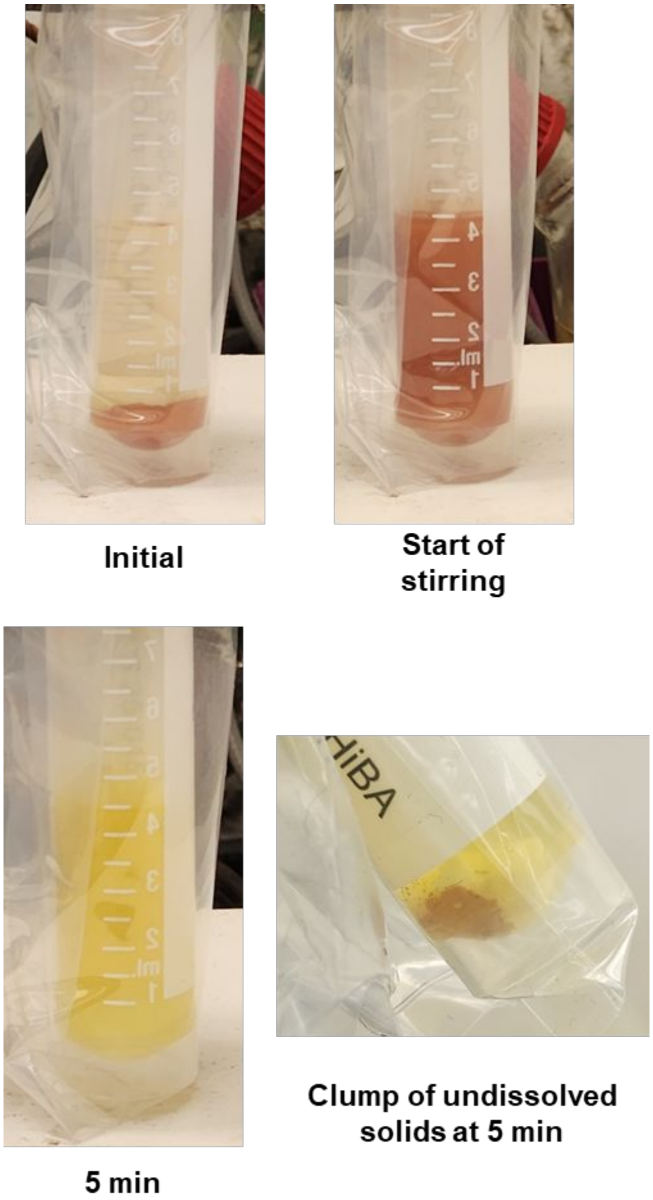

Figure S10. Photos showing the initial stages of the dissolution of ε-UO_3 in HNO_3-loaded 1.5 M DEHiBA in a polypropylene vial. The theoretical U concentration (assuming 100% dissolution and no organic phase volume change during dissolution) is 0.42 M.

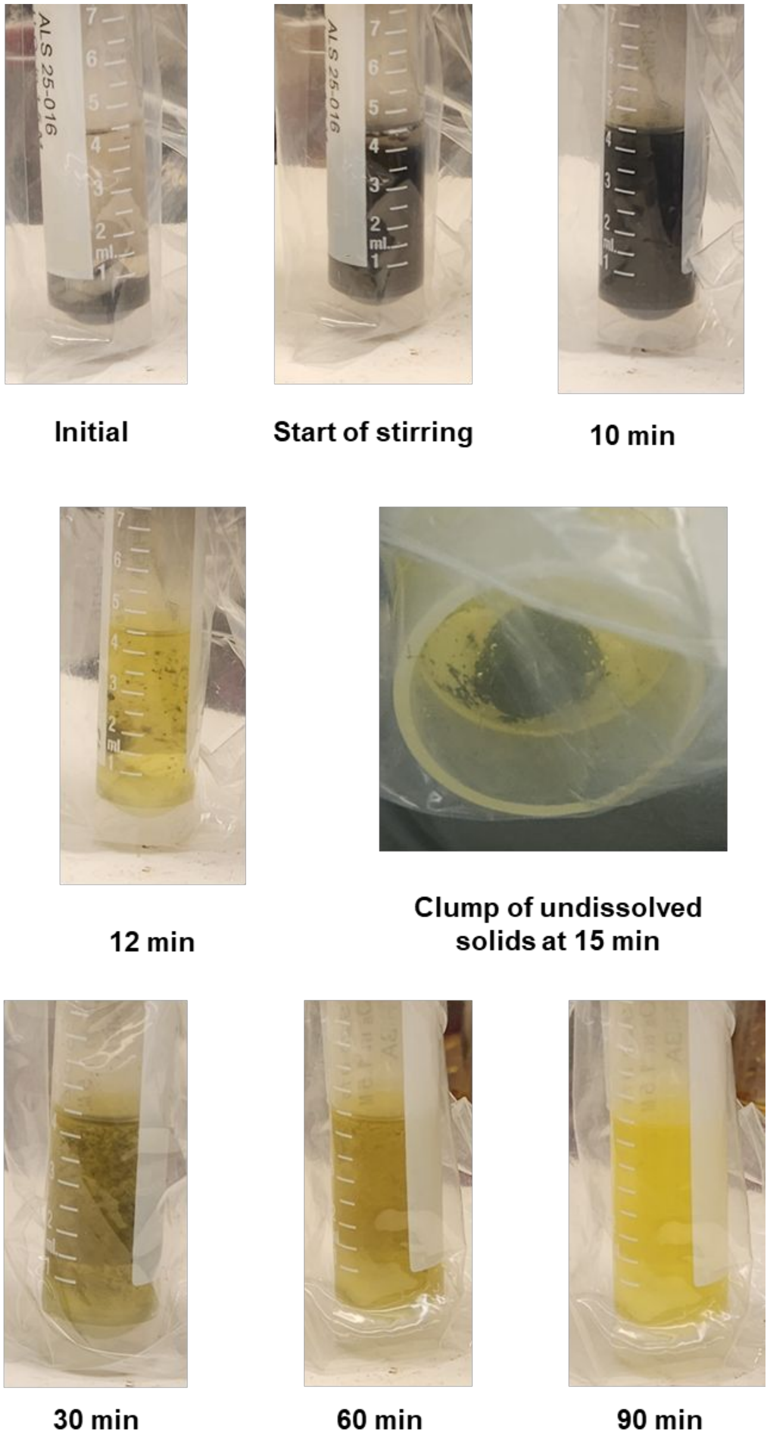

Figure S11. Photos showing the dissolution of α-U_3O_8 in HNO_3-loaded 1.5 M DEHiBA in a polypropylene vial. The theoretical U concentration (assuming 100% dissolution and no organic phase volume change during dissolution) is 0.42 M.

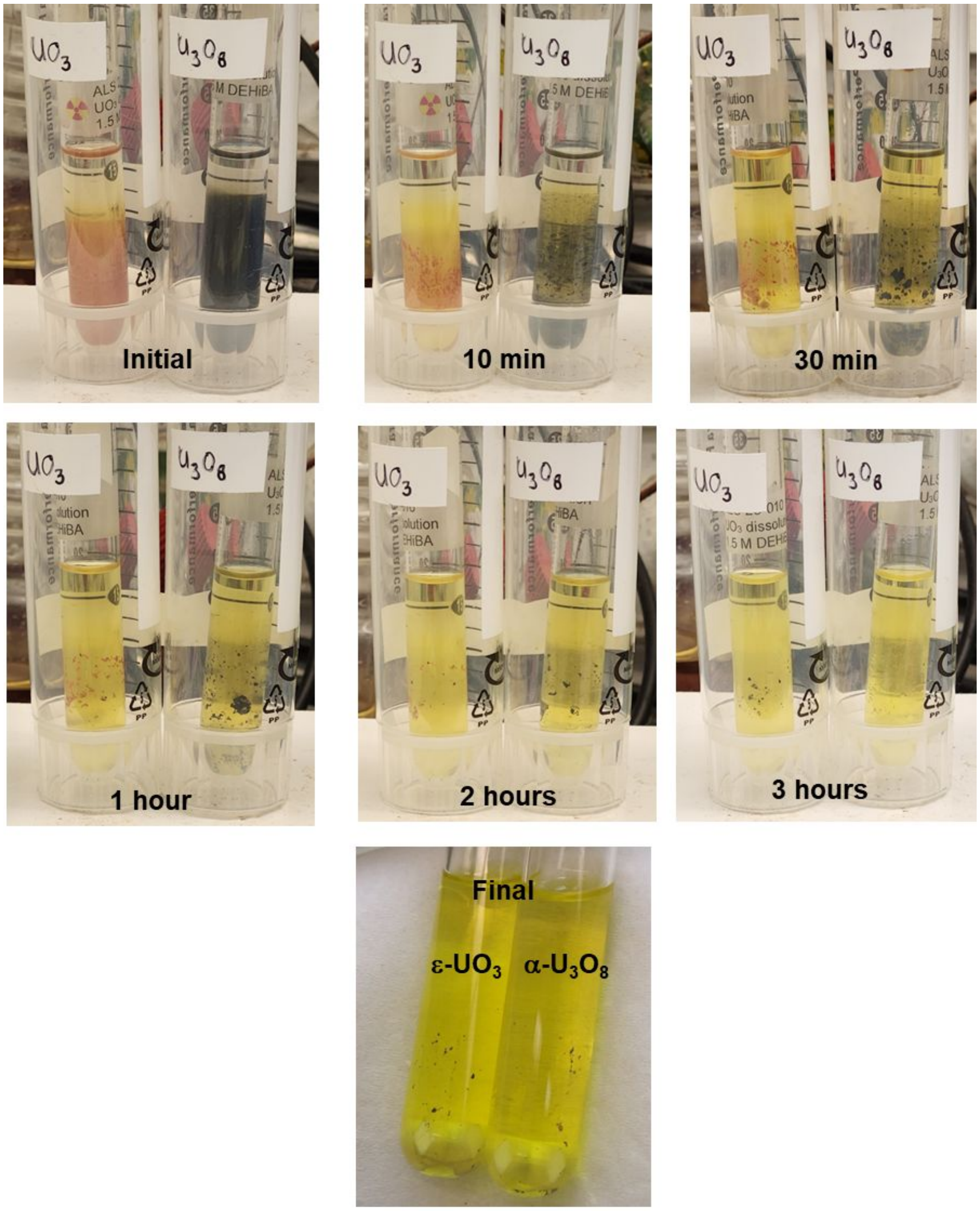

Figure S12. Photos showing the dissolution of ε-UO_3 (left) and α-U_3O_8 (right) in 1.5 M DEHiBA in a glass vial. The final reaction mixture after centrifuging is shown at the bottom. The theoretical U concentration (assuming 100% dissolution and no organic phase volume change during dissolution) was 0.42 M for both reactions. The colorless layer at the top of the vials at early timepoints suggests that (at least initially) the top ~20% of the solution does not mix well with the rest of the solution.

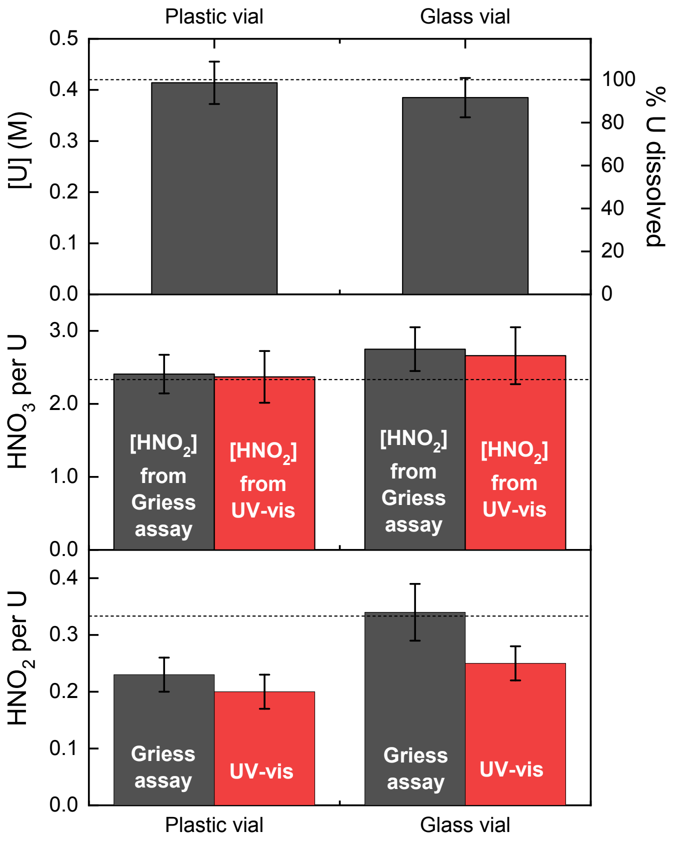

Figure S13. Results for reaction stoichiometry studies from dissolution of α-U_3O_8 in 1.5 M DEHiBA at a target U concentration of 0.42 M in a polyethylene vial and in a glass vial. (Top) U concentrations. The theoretical U concentration (assuming 100% dissolution and no volume change upon dissolution) is shown as a dashed line. (Middle) HNO_3 per U determined using the HNO_2 concentrations from Griess assays (black) and UV-vis (red). The theoretical value of 2.33 HNO_3 per U (equation 4 in the main text) is shown as a dashed line. (Bottom) HNO_2 per U determined by Griess assay (black) and UV-vis spectroscopy (red). The theoretical value of 0.33 eq HNO_2 per U (equation 4 in the main text) is shown as a dashed line.
